# Novel Strategies for Endothelial Preservation in Lung Transplant Ischemia-Reperfusion Injury

**DOI:** 10.3389/fphys.2020.581420

**Published:** 2020-12-18

**Authors:** Wolfgang Jungraithmayr

**Affiliations:** ^1^Department of Thoracic Surgery, University Hospital Freiburg, Freiburg, Germany; ^2^Department of Thoracic Surgery, University Hospital Zurich, Zurich, Switzerland; ^3^Department of Thoracic Surgery, University Hospital Rostock, Rostock, Germany

**Keywords:** lung, transplantation, ischemia-reperfusion injury, endothelium, strategies

## Abstract

Lung ischemia reperfusion (IR) injury inevitably occurs during lung transplantation. The pulmonary endothelium is the primary target of IR injury that potentially results in severe pulmonary dysfunction. Over the last decades, various molecules, receptors, and signaling pathways were identified in order to develop treatment strategies for the preservation of the pulmonary endothelium against IR injury. We here review the latest and most promising therapeutic strategies for the protection of the endothelium against IR injury. These include the stabilization of the endothelial glycocalyx, inhibition of endothelial autophagy, inhibition of adhesion molecules, targeting of angiotensin-converting enzyme, and traditional viral and novel non-viral gene transfer approaches. Though some of these strategies proved to be promising in experimental studies, very few of these treatment concepts made the transfer into clinical application. This dilemma underscores the need for more experimental evidence for the translation into clinical studies to invent therapeutic concepts against IR injury-mediated endothelial damage.

## Introduction

Ischemia reperfusion (IR) injury in lung transplantation (Tx) is associated with an increased rate of delayed graft function, but also acute and chronic graft rejection. As IR injury is unavoidably correlated with Tx, treatment strategies for the attenuation of IR injury should aim at protecting target compartments that are primarily affected by IR injury.

The pulmonary endothelium is the first site of IR injury. Already during organ ischemia, multiple donor-associated changes can alter the status of the pulmonary vasculature, that is e.g., the loss of surfactant, hypovolemia resulting in platelet occlusion of the vascular bed, an increase of pro-inflammatory mediators due to brain death, and an increase of adhesion molecules (Porteous and Lee, 2017). Upon reperfusion, the endothelial layer of the pulmonary vasculature produces reactive oxygen species (ROS) and other damaging agents (Preissler et al., 2011) which in turn results in endothelium swelling and detachment from the basement membrane (Pickford et al., 1990). This process results in a damage of the vascular permeability thereby facilitating leukocyte adherence and transmigration, the key step in mediating IR injury (Soroush et al., 2018). The interaction between leukocytes and endothelium depends on and involves a complex network of various proteins.

Over the last decades, a wide range of those proteins have been identified that mediate the differential steps necessary for the interaction between white blood cells and endothelium. Among them, selectins are responsible for a first contact and weak adhesion of leukocytes (Kawut et al., 2009), different adhesion molecules create a firmer contact of leukocytes on the endothelium and leukocyte extravasation across the endothelial wall (Muller, 2016), and finally the migration of leukocytes into the tissue is mediated by IL-8 and other mediators (Allen and Kurdowska, 2014). A number of other mediators such as TNFα, IL-1, IL-6, leukotriene B4, proteases, and elastases that are in part released from activated neutrophils, indirectly or directly, contribute to endothelial dysfunction and injury (Porteous and Lee, 2017).

Another target of IR that contributes to endothelial damage and tissue dysfunction after IR is the endothelial glycocalyx. This layer consists of proteoglycans and glycoproteins at the surface of the endothelium and harbors various chemokines, receptors, growth factors, and enzymes that play a central role in endothelial function. The endothelial glycocalyx has a pivotal importance in lung injury which has been shown during edema formation after lung IR injury when the integrity of the glycocalyx was damaged (Brettner et al., 2017).

During the last decades of research, many of these proteins, receptors, mediators, and inflammatory cascades participating in endothelial injury upon IR injury have been targeted by treatment strategies, either by antibodies, inhibitors, or modulators of the inflammatory milieu. Among these, older agents include the phosphodiesterase inhibitor cyclic AMP analog or nitride oxide.

We here will present and discuss the most recent and promising developments on strategies how to efficiently preserve the endothelium from lung IR injury.

### An Ambiguous Role for Autophagy Inhibition on Pulmonary Endothelium During IR Injury

Autophagy allows for the regular degradation and recycling of cellular components and thus promotes cell survival. However, upon injury, an imbalance in endosomal and autophagic pathways can enhance macromolecular or organelle degradation thereby causing cell death. Inhibition of autophagy was shown to result in reduced apoptosis indicating that the process of autophagy as a cell survival mechanism is rather endangering the lung during IR injury (Zhang et al., 2013). On the other hand, recent analyses on pulmonary microvascular endothelial cells exposed to hypoxia-reoxygenation revealed that the suppression of mitochondrial autophagy by 3-Methyladenine (3-MA), a selective PI3K inhibitor, inhibited the apoptosis of endothelial cells and enhanced its proliferation via the mechanistic Target of Rapamycin-pathway (Liu et al., 2019). Another study in human and mouse pulmonary microvascular endothelial cells convincingly showed that promoting autophagy with rapamycin ameliorated IR-induced lung edema and tissue inflammation due to injured endothelium (Zhang et al., 2015).

Taken together, research on targeting autophagy has so far been scarce and results presented seem to be ambiguous, so there is a clear need for more studies that proves an either endothelial-preserving or endothelial-damaging effect of autophagy.

### Stabilization of the Endothelial Glycocalyx From Degradation by Lung I/R Injury

**FIGURE 1:**
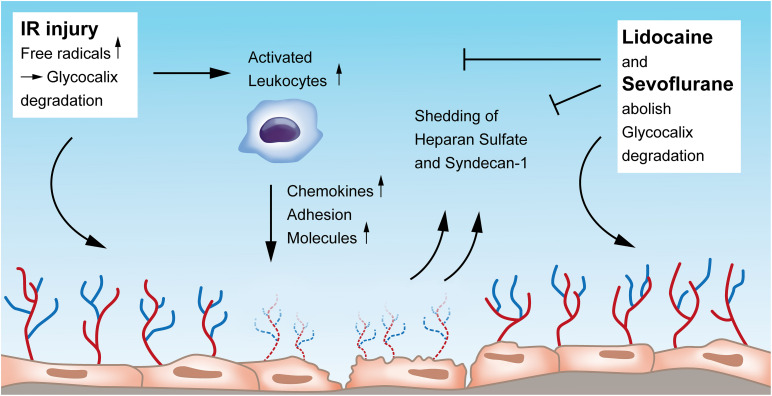
Endothelial glycocalyx degradation induced by ischemia-reperfusion injury and its restoration by Lidocaine and Sevoflurane. Upon IR injury, free radicals, among others, contribute to glycocalyx erosion. IR injury also leads to activation of leukocytes that in turn increase the secretion of chemokines and adhesion molecules, all damaging the endothelial layer. Heparan sulfate and syndecan-1 are shed into the blood when the glycocalyx is damaged by IR and could function as biomarkers of endothelial integrity. Both Lidocaine and Sevoflurane can restore and preserve damaged glycocalyx. IR, ischemia-reperfusion.

The luminal surface of vascular endothelial cells is lined with a glycocalyx, comprising of membrane-bound proteoglycans, glycosaminoglycans, and sialic acid–containing glycoproteins. This layer is pivotal for the integrity and functionality of the endothelium, as it modulates the interaction of the endothelium with the components of the circulating blood. The glycocalyx harbors a variety of chemokines, receptors, growth factors, and enzymes that play a central role in endothelial function. This layer protects the endothelium from shear stress which is caused by blood flow and serves as a vascular permeability barrier. Glycocalyx damage during IR can be detected by elevated plasma levels of e.g., heparan sulfate and syndecan-1, which suggests their use as biomarkers of endothelial integrity (Abassi et al., 2020) (Figure 1).

The glycocalyx of the pulmonary vasculature is thicker than that of other organs (Yang and Schmidt, 2013) which might be owed to the complex lung defense against internal and external antigens. During and after lung IR, the glycocalyx is degraded and leads to leukocyte activation and adhesion with subsequent pulmonary edema and lung injury. Experimental studies showed that the integrity of the glyocalyx layer could be preserved: e.g., in an auto-transplantation model, Rancan et al. (2018) could show that IR itself causes glycocalyx alterations with an increase in pulmonary edema and expression of adhesion molecules, and that by the administration of lidocaine, the described alterations of glycocalyx degradation could be abolished (Figure 1). The same research group proved in an *in vivo* auto-transplantation model of pulmonary IR that by preconditioning with sevoflurane, the pulmonary glycocalyx could be protected and the expression of leukocyte chemokines could be reduced (Casanova et al., 2016). In this study, authors compared the effects of sevoflurane with propofol on IR-injured endothelium and found that propofol, but not sevoflurane, induced glycocalyx destruction and a higher chemokine and adhesion molecule expression with also an increase of the glycocalyx components heparan sulfate and serum syndecan levels (Casanova et al., 2016). Work from Chappell et al. also revealed less shedding and a reduced leukocyte adhesion after sevoflurane pre-treatment in an isolated organ model (Chappell et al., 2010). In contrast, another group analyzed those endothelial glycocalyx-injuring markers heparan sulfate and human syndecan-1 in patients undergoing pulmonary resection with one lung ventilation, however, they could not find any difference in outcome between sevoflurane and propofol (Kim et al., 2018).

As reflected by increased research activity in recent years, the glycocalyx has gained more attention taking a pivotal role in the regulation of endothelial function and vascular permeability. Though the number of studies is limited, glycocalyx components such as syndecan-1 and heparan sulfate have the potential to serve as circulatory biomarkers for monitoring the severity of vascular endothelial damage during lung IR injury. Moreover, future research on glycocalyx protection and restoration has so far received a solid base, and pharmacologic and non-pharmacologic maneuvers on this topic should be promoted. Figure 1 provides an overview of the current understanding of the glycocalyx in the context of IR injury and its preservation by Lidocaine and Sevoflurane.

### Targeting P-selectin on I/R-Injured Pulmonary Endothelium

Selectins are molecules involved in the initial process of adhesion of activated neutrophils on the pulmonary endothelium. Early work has shown that when selectively blocking selectins with a monoclonal antibody, reperfusion-induced hyperpermeability was ameliorated (Demertzis et al., 1999; Levine et al., 2002). More recently, a particular role has been assigned to P-selectin in IR injury (Moore et al., 1985; Scalia et al., 1999). As a key endothelial cell rolling factor, P-selectin mediates the adhesion of leukocytes on the endothelium and binds to glycoprotein ligand-1 (PSGL-1) on leukocytes. Moore et al. (1985) showed that when inhibiting P-selectin by a monoclonal antibody, the increase of IR-induced permeability, lung sequestration of neutrophils, mononuclear leukocytes, and eosinophils could be significantly attenuated. But also the rolling and adhesion of platelets along arteriolar walls depends on P-selectin, suggesting that platelets are also responsible for contributing to the development of pulmonary IR injury (Roberts et al., 2004). In line with these data, recent work from Lam et al. (2018) confirmed the role of P-Selectin in interaction with PSGL-1 in the attenuation of pulmonary IR injury: when using recombinant human vimentin, leukocyte adhesion to endothelial and platelet monolayers where blocked on P-selectin-coated surfaces *in vitro*. *In vivo*, histologic findings of acute lung injury decreased.

These data underscore the relevant role of the adhesion molecule P-Selectin and its inhibition on pulmonary endothelium for the attenuation of IR injury.

### Immunotargeting of Angiotensin-Converting Enzyme and Antioxidant Enzymes Against Oxidative Injury

**FIGURE 2:**
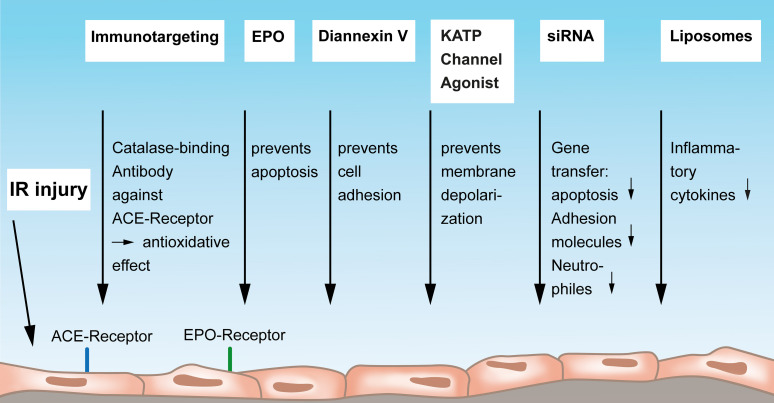
Mediators and strategies to protect the vascular endothelium from lung IR injury. Endothelial immunotargeting by a catalase-binding antibody to the angiotensin-converting-receptor on endothelium can reduce IR-reactive oxygen species. Erythropoietin exerts protective functions on endothelial cells by preventing apoptosis via endothelium-bound EPO receptors. Diannexin V mainly acts via preventing endothelial cell adhesion, and Kalium ATP-sensitive (ATP-sensitive potassium channels, KATP) channel agonists have the potential to prevent membrane depolarization thereby protecting the endothelium. Gene transfer of siRNA to the endothelium can reduced apoptosis, less production of adhesion molecules, and a reduced attraction of neutrophils. Finally, antibody-conjugated endothelial-targeted liposomes can inhibit the cytokine-induced inflammatory activation of the endothelium. IR, ischemia-reperfusion; ACE, angiotensin-converting enzyme; EPO, Erythropoetin; KATP, Kalium ATP-sensitive; siRNA, small interfering ribonucleic acid.

Angiotensin-converting enzyme (ACE) was previously identified as a cell-specific marker of endothelial injury (Atochina et al., 1997). Atochina et al. (1997) showed that IR induces significant elevation of ACE activity in the perfusate of isolated rat lungs. In this context, ACE serves as a surface endothelial antigen that drives leukocyte-mediated oxidative injury to endothelial cells. Muzykantov (2000) have shown that applying a monoclonal antibody against ACE serves as an affinity carrier for targeting of catalase to the pulmonary endothelial cells for specific augmentation of their anti-oxidative defense. The antioxidative effect of the conjugation of this ACE antibody to catalase has also been investigated and confirmed *in vivo* by Nowak and co-workers in a rat lung ischemia model (Nowak et al., 2007). They used the ACE monoclonal antibody (MAb) 9B9 and showed a better function of the affected lung but also decreased serum levels of endothelin-1 and less levels of inducible nitric oxide synthase. The same group evaluated the treatment of IR-injured lung allografts with conjugates of anti-ACE antibody with catalase (9B9-CAT) in an experimental set up of hypothermic preservation. Lungs that were conditioned with this antibody showed a significantly higher catalase activity and an anti-oxidative status after cold storage compared to the catalase treatment alone (Nowak et al., 2010). Finally, these authors extended their research on an anti-ACE catalase-binding antibody in a human model of isolated perfused and ventilated lung resection (Nowak et al., 2013) and thus provided a possibility of immunotargeting of human pulmonary endothelium toward a therapeutic approach in patients. Of note, in the research of antioxidant therapeutics against endothelial injury by reactive oxygen species, catalase was also conjugated to platelet/endothelial cell adhesion molecule-1 and was demonstrated *in vitro* as well as *in vivo* to have good protective effects against endothelial oxidative stress, injury, and lethality (Christofidou-Solomidou et al., 2003) (Figure 2). This approach could be confirmed by Kozower et al. (2003). The entire concept of vascular immunotargeting by a specific delivery of therapeutic agents to endothelial cells and by modulating enzymes conjugated with antibodies to endothelial surface molecules has been nicely summarized in a review by Shuvaev and Muzykantov (2011).

Taken together, these studies show that vascular immunotargeting of anti-oxidative enzymes, specifically conjugates to ACE, can effectively limit reactive oxygen species–mediated IR injury of the lung.

### Other Strategies to Protect the Vascular Endothelium From Lung IR Injury

This subsection will consider more therapeutic strategies on how to preserve and protect the vascular endothelium from IR injury that do not fall into the scope of the above described approaches (Figure 2). Erythropoietin (EPO) has been shown to positively influence acute lung injury caused by renal IR injury (Janmaat et al., 2010). EPO acts as the major regulator of erythropoiesis by stimulating growth, preventing apoptosis, and inducing differentiation of blood precursor cells. An explanation for a beneficial effect on lung injury could lie in the fact that endothelial cells express EPO receptors and thereby respond to EPO. Zhu et al. (2019) found in a model of remote lung injury induced by mouse renal injury that EPO exerts protective functions by promoting endothelial cell proliferation via the Janus Kinase-Signal Transducer and Activator of Transcription 3 pathway, rather than exerting antiapoptotic or anti-inflammatory mechanisms.

Sharma et al. (2018) analyzed the role of pannexin-1 (Panx1), a channel-forming glycoprotein that mediates signaling in vascular endothelium and plays a major role in the initiation of endothelial permeability, edema, leukocyte trafficking, and inflammation during lung IR injury. In an *in vivo* mouse lung hilar ligation model, authors found that endothelial pannexin 1 contributes to IR of the lung. Moreover, their results demonstrate a novel role of endothelial Panx1 channels in the regulation of vascular inflammation after lung IR: when blocking Panx1-mediated ATP release by Panx1 inhibitors, lung IR injury could be attenuated.

The inhibition of the adhesion of leukocytes and platelets to the endothelium was targeted in a study by Hashimoto and the thoracic research group at Toronto General Hospital. Their report in a rat lung transplant model says that Diannexin V, which is a homodimer of annexin V, ameliorates IR injury. Authors administered Diannexin V to the donor before procuring the lung and also to the recipient shortly after reperfusion and could prove a significantly enhanced graft function and an improved oxygenation. A mechanistic explanation for the observed benefits was that Diannexin is designed to shield phosphatidylserine, to prevent cell adhesion, to improve blood flow and to diminish subsequent tissue injury (Hashimoto et al., 2016). Practically, authors concluded to employ this compound at an early phase after transplantation to the recipient.

The group around Chatterjee, Christy and Fisher, all renowned scientists in shear stress-related mechanosignaling research in lung ischemia provided an excellent review article showing that the mechanosignaling cascade during lung IR injury is initiated by a mechanosensing complex, transmitted by endothelial cell membrane depolarization due to closure of KATP (ATP-sensitive potassium channels) channels, resulting in activation of NADPH oxidase, NOX2 with generation of ROS and increased intracellular Ca2^+^ (Chatterjee et al., 2014). Authors point to an approach to prevent the mechanosignaling cascade by using a KATP channel agonist to prevent endothelial cell membrane depolarization. Although these agents are available for this purpose, they have not yet been trialed clinically (Chatterjee et al., 2014).

**FIGURE 3:**
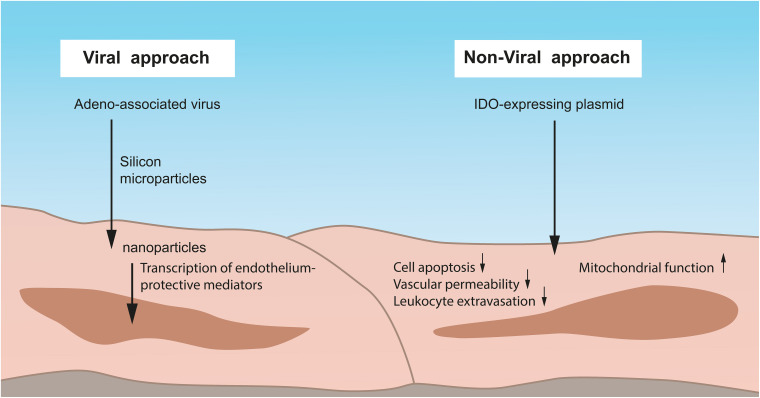
Viral and non-viral approaches to preserve IR-injured lung endothelium. One of the most commonly employed viral vectors is the adeno-associated virus. By such a virus, silicon microparticles can be delivered into inflammation-associated endothelium where it is internalized with the subsequent release of therapeutic nanoparticles. The virus becomes free within the cytoplasm, undergoes genetic cargo, and transfers into the nucleus for the transcription of endothelium-protective mediators. In contrast, non-viral approaches have advantages over the commonly used viral systems such as lower immunogenicity, reduced inflammation, and an overall better safety. A human Indoleamine-2,3-dioxygenase (IDO)–expressing plasmid can be delivered by a specific promoter into the endothelium and reduces cell apoptosis, vascular permeability, and leukocyte extravasation. Moreover, the mitochondrial function and ultrastructure of the endothelium is protected from oxidative stress. IDO, Indoleamine-2,3-dioxygenase.

Viruses are used since long as gene delivery vectors to treat diseases by therapeutic gene expression (Waehler et al., 2007) (Figure 3). One of the most commonly investigated viral vectors is the adeno-associated virus. As an immunotherapeutic approach, McConnell et al. (2014) took advantage of such a virus as a “trojan horse” that delivers silicon microparticles into inflammation-associated endothelium where it is internalized and subsequently releases therapeutic nanoparticles. The virus than becomes free within the cytoplasm undergoing genetic cargo to successfully transfer into the nucleus for transcription. This kind of uptake is independent from cell receptors, enabling access to not only endothelial cells. Authors, thus, showed that whole organs can be genetically modified using this particle platform as a technique for improving organ or vessel transplant.

But also a non-viral gene transfer approach for the preservation of IR-injured endothelium is possible and, beyond that, provides several advantages over the commonly used viral systems, such as lower immunogenicity, reduced inflammation, and an overall better safety. Using this approach, Liu and colleagues analyzed the role of Indoleamine-2,3-dioxygenase (IDO) as a cytosolic enzyme possessing both immune modulating and antioxidant properties against lung transplant IR injury (Figure 3). They delivered a human IDO (hIDO)-expressing plasmid driven by an endothelial cell-specific endothelin-1 promoter into rat donor lungs and found that hIDO expression specifically enhanced in endothelial cells within lung grafts and prevented endothelial cell apoptosis, reduced vascular permeability and leukocyte extravasation *in vivo* (Liu et al., 2019). *In vitro*, they showed that increased IDO activity in endothelial cells protected its mitochondrial function and ultrastructure from oxidative stress through stabilization of the intracellular redox status (Liu et al., 2019) (Figure 3). Authors, thus, presented a strategy of non-viral gene transfer for the protection of lung endothelium, at the same time avoiding the toxic effects of a traditional viral therapy.

Another promising treatment tool is siRNA (Figure 2). By using this highly specific technology, an organ-specific gene transfer can be achieved even without the need of viral vectors or other transfection agents. Zhang et al. (2004) showed that by intranasal delivery of siRNA into mouse IR-injured endothelial cells, apoptosis can be attenuated via an overexpression of Heme oxygenase-1 (HO-1), an important cytoprotective enzyme in IR injury. This concept contributes to the identification of genes that modulate IR-induced apoptosis with potential therapeutic relevance. siRNA was also used to target adhesion molecules in an *in vitro* work by Walker et al. (2011). They transfected human lung microvascular endothelial cells with specific siRNA and found that not only adhesion molecules on the endothelium were decreased but also the adhering neutrophils were diminished (Walker et al., 2011) (Figure 2).

Another drug delivery system to target IR-injury–mediated reactive oxygen species in the vascular endothelium are liposomes. Howard et al. (2014) designed endothelial-targeted liposomes carrying a potent superoxide dismutase (SOD)/catalase mimetic, EUK-134, antibody-targeted against PECAM. The Ab/EUK/liposome conjugates bound to endothelial cells and inhibited cytokine-induced inflammatory activation *in vitro* (Figure 2); *in vivo*, they accumulated in lungs after intravascular injection and protected against pulmonary edema in endotoxin-challenged mice (Howard et al., 2014). The advantage of this compound is that it is already in clinical use.

Oxidized phospholipids are known to enhance the endothelial barrier by maintaining the endothelial cell-cell junctions. Such a compound, OxPAPC, was shown to directly bind to different cytoskeletal proteins thereby stabilizing the cytoskeletal reorganization and endothelial cell barrier enhancement and, thus, the vascular integrity (Birukova et al., 2014).

## Summary and Conclusion

Maintenance of the integrity of the pulmonary vascular endothelium is pivotal in order to protect from IR injury in lung transplantation. A number of therapeutic strategies with strong impact have been developed, among them are mediators against ROS and inhibition of endothelial-related adhesion molecules to mainly attenuate neutrophil transmigration into the pulmonary tissue. But also very promising recent approaches, e.g., the stabilization of the endothelial glycocalyx seems to be important as this layer is the prominent interface between the blood stream, its constituents, and the vascular endothelium. On the other hand, novel developments such as the concept of the inhibition of endothelial autophagy are promising, but need more experimental work in order to gain solid scientific evidence. Finally, viral as well as non-viral strategies and siRNA treatment approaches are emerging and seem fascinating. At the same time, they have fewer side effects in contrast to immunotargeting approaches. While the concept of liposome release is already in clinical use, the majority of the other approaches did not translate into clinics up to now. We can only speculate why these approaches did not reach clinical application. One reason is certainly that there is a clear need for more translational research to clarify a definite role for these therapeutic tools in endothelial preservation. Another reason could lie in the fact that there is more support needed from respective pharmacological industrial partners to apply these measures broadly into the clinic. The future, in my eyes, lies in making major efforts in the prevention of the initiating damaging effects of IR injury such as preserving the glycocalyx, rather than the treatment of the endothelium that is already IR-injured. The impact of lung endothelium IR-injury on the development and incidence of transplant rejection and overall transplant outcome is undoubted, therefore, major efforts need to be taken to strengthen those promising experimental therapeutic approaches and translate them into clinical trials.

## Author Contributions

The author confirms being the sole contributor of this work and has approved it for publication.

## Conflict of Interest

The author declares that the research was conducted in the absence of any commercial or financial relationships that could be construed as a potential conflict of interest.
